# Education review articles – a call for advanced courses in Medical Imaging and Radiation Oncology Physics

**DOI:** 10.1002/acm2.12680

**Published:** 2019-07-19

**Authors:** Michael Mills

**Affiliations:** ^1^ Radiation Oncology University of Louisville Louisville KN USA

Surveying the landscape of the educational experience of medical physicists, there are many relatively recent accomplishments, and our educational organizations have been active promoting some necessary improvements. Maintenance of Certification became a reality in the early years of this century. The requirement to complete a residency program to sit for the American Board of Radiology (ABR) certification examinations is a reality as of 2014. The Commission on Accreditation of Medical Physics Education Programs (CAMPEP) has accredited over 50 academic programs, over 100 residency programs in therapy physics and over 25 residency programs in imaging physics. The majority of these programs were first accredited in this decade. Additionally, the American Association of Physicists in Medicine (AAPM) Education Council and the Society of Directors of Academic Medical Physics Programs have been active promoting medical physics education, encouraging quality, providing for coordination between education programs and establishing best practices in medical physics education. The AAPM has hundreds of Councils, Committees, Subcommittees, Task Groups and Working groups, all involved in some aspects of the evolving nature of technology, clinical practice, and the knowledge base of our profession.

Even with all of this activity, however, keeping up with changes in the field and providing for contemporary education is a daunting task and challenging to do well. Continuing education is absolutely necessary in this profession. If we fail to keep up, our practice skills can become obsolete in a few years. So as a profession, we face two challenges in education: how do we bring students up to a baseline level of practice in our academic and residency programs, and how do we maintain a level of practice proficiency? This editorial discusses one aspect of the first challenge.

Respecting CAMPEP academic programs, there is only one full structured course required in radiation oncology physics (at the level of the Khan or Hendee text) and one required in imaging physics (at the level of Bushberg). These courses, usually one semester each, can do little more than provide the most basic of introduction to the science of the field. Thus, a growing knowledge gap exists between the material in these courses and the information published each month in our scholarly journals, the *JACMP* and *Medical Physics*. An argument easy to make is that an advanced structured course in both radiation oncology and imaging physics of one semester each would be not only desirable, but perhaps essential for the training of the next generation of medical physicists. But what would such a course look like?

We envision the second course in therapy physics to be site/disease based and would focus on integrating the technology with the clinical challenges we face each day. As an example, consider prostate cancer. Three or four lectures would include the cancer biology and progression, staging and scoring, treatment approaches, planning and optimization algorithm details, Image Guided Radiation Therapy (IGRT), outcomes, and avenues for research. Other disease sites, including Central Nervous System (CNS), Gynocology (GYN), brain, lung, head and neck, and others would follow similarly. An advanced course in imaging physics would deal with specific sites (lung, mammography, CNS, pelvis, and others), using the latest insights into use of advanced techniques such as molecular imaging, dual‐energy imaging, image registration, artificial intelligence, computer‐aided diagnosis and other emerging technologies.

The justification for such courses is that this is the type of work therapy and imaging physicists face every day. It is our calling to solve imaging problems for specific disease sites and to assure knowledgeable and precise clinical practice respecting therapy interventions. Yet so often, entry‐level medical physicists are less prepared than they could be to undertake these challenges.

The basis for these advanced courses would include, among other references, a series of review articles published in a public forum. The *JACMP* would be an ideal place to publish such articles since the journal is open access and the author retains ownership of the copyright. There would therefore be no restriction on the use in an educational setting as the articles are free to access and use. These review articles would be peer reviewed and would form the basis for the second advanced course in therapy or imaging physics.

Since the *JACMP* has an established category to publish Education Articles, we are issuing a call for the community to volunteer its time to create and publish Education Review Articles as described. To avoid duplication of effort, please email the Editor and volunteer your article for a therapy or imaging site along with a timetable for submission. We believe these articles will be very valuable to the community and essential to prepare medical physics students and residents for clinical practice.

